# Tumor necrosis factor-like weak inducer of apoptosis regulates quadriceps muscle atrophy and fiber-type alteration in a rat model of chronic obstructive pulmonary disease

**DOI:** 10.1186/s12971-017-0148-5

**Published:** 2017-11-09

**Authors:** Jun-Juan Lu, Qing Wang, Li Hua Xie, Qiang Zhang, Sheng Hua Sun

**Affiliations:** 1 Department of Respiratory Medicine, The Third XiangYa Hospital of Central South University, 138 Tongzipo Road, Changsha, Hunan 410013 People’s Republic of China; 2 Department of Respiratory Medicine, Changsha Central Hospital, Changsha, Hunan 410004 People’s Republic of China

**Keywords:** Chronic obstructive pulmonary disease, Quadriceps muscle atrophy, Fiber-type alteration, Tumor necrosis factor-like weak inducer of apoptosis, Muscle ring finger-1, Peroxisome proliferator-activated receptor-γ coactivator-lα, Nuclear factor-κ B

## Abstract

**Background:**

In chronic obstructive pulmonary disease (COPD), weakness and muscle mass loss of the quadriceps muscle has been demonstrated to predict survival and mortality rates of patients. Tumor necrosis factor (TNF)-like weak inducer of apoptosis (TWEAK), as a member of the TNF superfamily, has recently been identified as a key regulator of skeletal muscle wasting and metabolic dysfunction. So our aim was to study the role of TWEAK during quadriceps muscle atrophy and fiber-type transformation in COPD model rats and its possible pathway.

**Methods:**

Forty-four healthy male adult Wistar rats were randomly divided into two groups: A normal control group (*n* = 16) and a COPD model group (*n* = 28). The COPD group was exposed to cigarette smoke for 90 d and injected with porcine pancreatic elastase on day 15, whereas the control group was injected with saline alone. Following treatment, weights of the quadriceps muscles were measured and hematoxylin and eosin staining was performed to identify structural changes in lung and quadriceps muscle tissue. Immunohistochemical staining was also conducted to determine the localization of TWEAK, nuclear factor (NF)-κB, muscle ring finger (MuRF)-1 and proliferator-activated coactivator (PGC)-1a proteins in the quadriceps muscle, and western blotting was used to assess the level of protein expression.

**Results:**

Compared with controls, COPD model rats exhibited significantly lower quadriceps muscle weight (*P* < 0.05) accompanied by fiber atrophy and disordered fiber arrangement, a wide gap between adjacent muscle fibers, a significant reduction in nuclear number (*P* < 0.05) and an uneven size distribution. The proportion of fiber types was also significantly altered (*P* < 0.05). In addition, TWEAK expression in the quadriceps muscle of COPD model rats was significantly higher than that in control rats (*P* < 0.05), and was significantly associated with quadriceps atrophy and fiber-type alteration (*P* < 0.05). Levels of NF-κB, MuRF1 and PGC-1α expression also significantly differed between the two groups (*P* < 0.05).

**Conclusions:**

Collectively these data suggest that increased levels of TWEAK may lead to skeletal muscle atrophy and fiber-type alteration, which in turn may be associated with activation of the ubiquitin-proteasome pathway, involving NF-κB, MuRF1 and PGC-1α as potential regulatory factors. These preliminary results in rats suggest that TWEAK may be a therapeutic target for the treatment of muscle atrophy in COPD.

## Background

Chronic obstructive pulmonary disease (COPD) is a lung disorder characterized by progressive airflow obstruction, due to chronic pulmonary inflammation, and airway remodeling that typically involves the development of emphysema. It has been predicted that COPD will be the third leading cause of death worldwide by 2020 [1]. Comorbidities, particularly skeletal muscle dysfunction with or without muscle loss, are characteristic in patients with COPD, even during early stages of the disease [2]. Muscle dysfunction is defined as the impairment of muscle strength or endurance, as the two main properties of muscles [2]. In COPD, weakness (defined as reduced muscle force) and muscle mass loss of the quadriceps muscle have been demonstrated to predict the survival and mortality rates of patients [2, 3]. Previous studies have also indicated that COPD patients have a reduced proportion of type I fibers, which in turn is associated with disease severity [2–5]. Several factors have been implicated in the etiology of COPD muscle dysfunction, including oxidative stress, systemic inflammation, structural abnormality, mitochondrial derangement, autophagy, muscle wasting and deconditioning [1, 6–17]. However, the underlying mechanisms of quadriceps muscle atrophy and fiber-type alteration in patients with COPD remain unknown.

Cigarette smoking is among the most common causes of COPD, as demonstrated in mice exposed to cigarette smoke daily for 8 weeks, whereby a 157% increase in serum tumor necrosis factor (TNF)-α, accompanied by significant decreases in the levels of peroxisome proliferator-activated receptor γ co-activator 1α (PGC-1α) mRNA within soleus and extensor digitorum longus muscles were observed [18]. In addition, in smoker patients with advanced COPD, biopsies of vastus lateralis muscle identified reduced levels of oxidative markers and regulators, including citrate synthase and PGC-1α, in 23% of COPD patients, relative to smokers without COPD [19]. Elevated levels of muscle TNF-α mRNA were also observed alongside the reductions in oxidative markers, although TNF-α protein was undetectable. Thus, the roles of TNF-α in COPD-related muscle wasting remain unclear. TNF-like weak inducer of apoptosis (TWEAK), as a member of the TNF superfamily, has recently been identified as a key mediator of skeletal muscle wasting and metabolic dysfunction [19]. Previous studies have demonstrated that the TWEAK/fibroblast growth factor-inducible (Fn)-14 system mediates skeletal muscle wasting in disuse conditions and in response to starvation [19, 20]. Furthermore, it has been documented in vitro and in vivo that increased levels of TWEAK may inhibit the regenerative properties of skeletal muscle, through regulatory effects on the self-renewal of satellite cells and proliferation, fusion and differentiation of myoblasts into multinucleated myotubes [21–24]. However, TWEAK may regulate COPD-related muscular atrophy through a number of potential mechanisms, which are yet to be determined in patients or animal models.

In many instances, acute loss of muscle mass is dependent on increased breakdown of muscle protein, mediated by the ubiquitin (Ub) 26S–proteasome system [1]. During acute muscle atrophy, the rate-limiting enzymes associated with the loss of muscle mass include the Ub-E3 ligation enzymes atrogin-1/muscle atrophy F-box and MuRF1. The genetic deletion of these “atrogenes” attenuates muscle atrophy under various conditions, for example, sepsis-induced atrophy, ventilation-induced atrophy hydrogen peroxide-induced atrophy and so on [25, 26], and increased expression of MuRF1 and atrogin-1 has been documented in the quadriceps muscle during COPD [27]. In addition, previous results suggest that PGC-1α serves a key role in preserving skeletal muscle mass and mitochondrial content under atrophic conditions [28]. It has also been documented that the progressive muscle atrophy observed in TWEAK-transgenic (Tg) mice is significantly attenuated in TWEAK-PGC-1α double Tg mice, suggesting that PGC-1α serves an important role in TWEAK-induced muscle atrophy [29]. Nuclear factor (NF)-κB is a primary mediator of the cellular response to inflammatory stimuli [30], and has been implicated in the transcriptional regulation of atrogin-1 and MuRF1 [31]. Thus, TWEAK may alter quadriceps atrophy and fiber-type transformation in COPD through regulation of NF-κB and its potential downstream effectors, namely PGC-1α and MuRF1.

Whereas the levels of several classical cytokines associated with muscle wasting (such as TNF-a, insulin-like growth factor-1, hypoxia inducible factor 1, and mammalian target of rapamycin) are increased in COPD, the role of TWEAK in muscle mass loss during COPD remains unknown [1, 4, 7, 8]. Therefore, the present study was to determine the potential roles of TWEAK during quadriceps muscle atrophy and fiber-type transformation in COPD model rats and its possible pathway. Potential associations between TWEAK, NF-κB, MuRF1 and PGC-1α in the pathology of COPD were also investigated.

## Methods

### Animals

A total of 44 male Wistar rats (age, 8 weeks) weight, 180-220 g) were used in the present study. The rats were purchased from the Agricultural University of Hunan (Changsha, HuNan, China) and were housed in a pathogen-free, temperature- and humidity-controlled environment (70% humidity; 20 ± 2 °C) under a 12/12 h light/dark cycle in the Third Xiangya Hospital Experimental Animal Center of Central South University (Changsha, Hunan, China). Animals were tested periodically to ensure that they remained pathogen-free. For biochemical assays, the rats were randomly sorted into a control group (*n* = 16) and a COPD group (*n* = 28). Rats in the COPD group were exposed to cigarette smoke (CS) from commercial filter cigarettes (Leiothrix cigarettes, 8 mg of tar and 0.6 mg of nicotine per cigarette; Changsha Cigarette Factory, Changsha, China) for 90 d and on day 15 were exposed to porcine pancreatic elastase (Sigma-Aldrich; Merck KGaA, Darmstadt, Germany) via tracheal dropping following tracheotomy, whereas the control group was injected with saline alone. Rats in the COPD group were exposed to the smoke emitted from 20 burning cigarettes per day, as described previously by Menegali et al. [32]. Briefly, animals were placed in a covered inhalation chamber (60 × 70 × 80 cm) and positioned under an exhaust hood. A cigarette was coupled to a plastic 60-ml syringe so as to draw in and expelled cigarette smoke into the exposure chamber. A total of 1 l smoke (20 puffs of 50 ml each) was aspirated from each cigarette and each puff was immediately injected into the inhalation chamber. When 1 L of smoke had been injected into the chamber, animals were maintained in this condition (3% smoke) for 6 min. The cover was then removed from the inhalation chamber and the exhaust hood switched on in order to remove the smoke within 60s. This process was immediately repeated. Animals were sacrificed in the day 90. Samples were isolated from all animals in each group for histological analysis. The right ventricle was perfused with sterile saline (0.9%) to remove blood from the lung. The right lung was fixed by infusion with 4% phosphate buffered formalin (pH 7.2) in 25 cm H_2_O for 2 min at 4 °C through a tracheal catheter, after which it was removed and weighed. The right lungs were then fixed in 4% paraformaldehyde at room temperature for 24 h and embedded in paraffin. Serial sagittal sections (5 μm) were obtained for histological and morphometric analyses. Samples of left lung tissue and quadriceps muscle were stored at −70 °C for no more than a month for later experiment use. The study was approved by the Institutional Review Board of Central-South University and conformed to the guiding principles for research involving animals and human beings [33].

### Tests of pulmonary function

A small animal lung function instrument (PLY3211; Buxco Research Systems, Wilmington, NC, USA) provided by the School of Basic Medical Science of Central South University (Changsha, China), was used to measure pulmonary function in the rats. Briefly, at day 90 rats were weight and subsequently anaesthetized by an intraperitoneal injection of 10% chloral hydrate (3 ml/kg; Ming Bo Biological Technology Co., Ltd., Shanghai, China) and maintained under a deep plane of anesthesia. The trachea was opened with an inverted Y-shaped incision at the second and third cartilage ring and immediately intubated with a Y-type cannula. Inspiration and expiration volumes of the lungs were then measured. An outlet of the intra-tracheal Y-type cannula was connected to a pressure transducer linked to a pulmonary mechanics analyzer (PLY3211; Buxco Research Systems), and the other was used for administration of air into the lungs. A total of 6.0 ml air was administered into the trachea and the forced expiratory volume at 0.3 s (FEV0.3), forced vital capacity (FVC), peak expiratory flow (PEF) and ratio of FEV0.3/FVC were measured by the analyzer.

### Evaluation of the model

The evaluation criteria used to determine whether establishment of the COPD rat model was successful were based on measurements of weight change, hematoxylin and eosin (H&E) staining of lung tissue and lung function, as described previously [34].

### Immunohistochemical staining

To block endogenous peroxidase activity, 5 μm-thick deparaffinized sections were incubated with 1% H_2_O_2_ for 30 min at room temperature. To observe the morphological characteristics and detect protein expression in the quadriceps muscle, the quadriceps muscle tissues were reacted overnight at 4 °C with anti-TWEAK (ab37170; 1:400; Abcam, Cambridge, UK), anti-NF-κB (sc8008; 1:300; Santa Cruz Biotechnology, Inc., Dallas, TX, USA), anti-PGC-1α (bs-1832R, 1:300, BIOSS, Beijing, China) and anti-MuRF1 (bs-2539R; 1:300; BIOSS, Beijing, China) antibodies. Samples were then washed with PBS and incubated with corresponding horseradish peroxidase-conjugated secondary antibodies (PV-8000; 1:200; ZSGB-BIO, Beijing, China) for 1 h at room temperature. Following the removal of non-reacted secondary antibodies by washing with PBS, samples were incubated with 3,39-diaminobenzidine (DAB; Sigma-Aldrich; Merck KGaA) in a DAB-4HCl-H_2_O_2_ solution to visualize immunolabeling. Some sections were also counterstained with hematoxylin and eosin and mounted with a coverslip with neutral resins. Immunohistochemical analysis was performed using Image-Pro Plus 6.0 software (Media Cybernetics, Inc., Rockville, MD, USA). A total of 4–5 images that were positive for protein expression were randomly selected and their integral luminosity values and average optical density were assessed using a light microscope.

### ATPase histochemical staining (calcium-cobalt method)

The calcium-cobalt method developed by Padykula & Herman [35] was modified to improve the buffering capacity of the medium. Briefly, quadriceps muscle sections were fixed for 2 min in cacodylate-buffered 4% formaldehyde at pH 7.0. No fixation would have lead to sections floating off the coverslip while prolonged fixation would have affected enzyme reactivity [36, 37]. Sections were incubated for 20 min at 37 °C in a freshly-made medium consisting of 8 ml tris-(hydroxymethyl)-aminomethane (1.0 M), 4 ml calcium chloride (0.18 M) and 60 mg ATP disodium salt, which was made up to 30 ml in distilled water and adjusted to pH 9.5 with 0.1 N-HCl before being brought up to a final volume of 40 ml). Thus the final concentration of ATP was 2.4 mM. Following two washes in distilled water, sections were immersed in 2% cobalt chloride for 3 min, then washed again twice in distilled water and developed in dilute ammonium sulfide for 1 min at room temperature, and then were assessed using a light microscope and analyzed with Image-Pro Plus 6.0 software (Media Cybernetics, Inc., Rockville, MD, USA).

### Western blotting

Quadriceps muscle homogenates lysed in a tissue lysis buffer (50 mM Tris, pH 8.0; 5 mM EDTA; 150 mM NaCl; 1% nonionic detergent; 0.5% sodium deoxycholate; and 0.1% sodium dodecyl sulfate) and a protease inhibitor cocktail (Sigma-Aldrich; Merck KGaA) for 10 min at 4 °C. Lysates were centrifuged at 13000 x *g* for 15 min at 4 °C. Bicinchoninic acid assay protein quantification kit (Wellsbio Inc., Changsha, China) was used for protein measurement. Protein (30–60 μg) was mixed 1:1 with SDS loading buffer (20% glycerol, 4% SDS, 3.12% dithiothreitol, 0.2% bromophenol blue, and 0.1 mol/l Tris HCl, pH 6.8, all from Sigma-Aldrich; Merck KGaA), and incubated at 100 °C for 4 min. A total of 50–100 μg protein was loaded per lane and separated by 10% SDS–PAGE and transferred onto a polyvinylidene difluoride microporous membrane (Millipore, Billerica, MA). Membranes were incubated with diluted primary antibodies TWEAK(ab37170,1:200,Abcam, Cambridge, UK), NF-κB(sc8008,1:400,Santa Cruz Biotechnology, Inc., Dallas, TX, USA), PGC-1α and MuRF1 (bs-2539R,1:200,BIOSS, Beijing, China), β–actin (60008–1-Ig,1:4000,Proteintech, Chicago, IL, USA), overnight at 4 °C and washed three times with TBST. Membranes were subsequently incubated with secondary anti-rat antibody with horseradish peroxidase conjugate (00001–9; 1:3000; Proteintech Group, Inc., Chicago, IL, USA) for 1 h at room temperature and washed again. Protein expression was measured by immunoblotting. Immunoreactivity was detected by enhanced chemiluminescence substrate (Beyotime Institute of Biotechnology, Haimen, China). Band densities were determined using an imaging densitometer and were analyzed with Quantity One v4.62 software (Bio-Rad Laboratories, Inc., Hercules, CA, USA). Protein expression was corrected with β–actin.

### Statistical analysis

Data are presented as the mean ± standard error of the mean. Comparisons of physiological, clinical, molecular and structural variables between the two study groups were made using Student’s t-test. Correlations between clinical, physiological and biological variables were determined using the Pearson’s correlation coefficient between groups. All data were analyzed using SPSS 18.0 software for Windows (SPSS Inc., Chicago, IL, USA) and *P* < 0.05 was considered to indicate statistical significance.

## Results

### Evaluation of COPD rat model

**Fig. 1:**
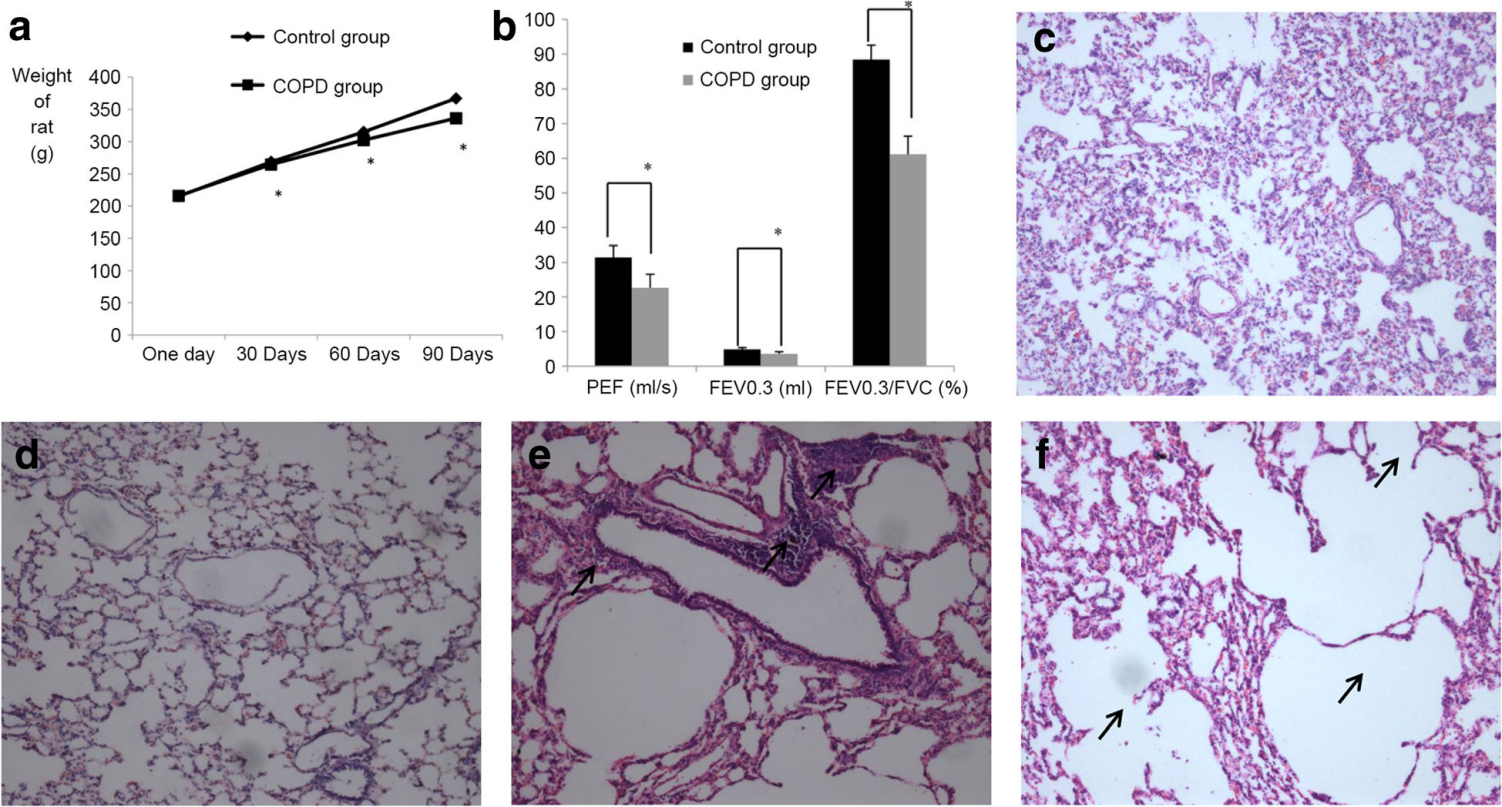
Evaluation of COPD rat model. **a** Weight change of healthy control and COPD groups over the 90-day test period. At 30, 60 and 90 days, the weights of COPD rats were significantly lower than that of controls. *n* = 10, *P < 0.05,versus the control group. **b** Rat lung function. The PEF, FEV0.3 and FEV0.3/FVC values of COPD rats were also significantly reduced compared with controls. n = 10, *P < 0.05,versus the control group. **c-f** Hematoxylin and eosin staining of rat lung tissue (magnification, ×100). **c** and **d** The lung tissue of control rats exhibited thin alveolar septa, normal alveoli and no inflammatory cell infiltration. **e** and **f** The lungs of COPD rats exhibited damaged alveolar septa, alveolar enlargement and inflammatory cell infiltration (indicated by black arrows). *P < 0.05. COPD, chronic obstructive pulmonary disorder; PEF, peak expiratory flow; FVC, forced vital capacity; FEV0.3, forced expiratory volume at 0.3 s

To determine whether the COPD rat model was successfully established, the weight, lung function and lung histomorphology of rats were measured. All rats survived until the end of the 90-day experimental period. Relative to control rats, the body weights of COPD rats increased at a significantly slower rate (*P* < 0.05; Fig. 1a).

In measuring parameters of lung function, it was observed that the PEF, FEV0.3 and FEV0.3/FVC of COPD rats were significantly decreased in the COPD group relative to controls (31.40 ± 3.34 vs. 22.69 ± 3.88 ml/s, *P* < 0.05; 4.88 ± 0.49 vs. 3.60 ± 0.58 ml, *P* < 0.05 and 88.41 ± 4.17 vs. 61.18 ± 5.19%, *P* < 0.05, respectively; Fig. 1b).

Based on histological analysis, lung tissue samples obtained from control rats (Fig. 1c and d) exhibited thin alveolar septa, normal alveoli and no inflammatory cell infiltration, whereas those obtained from COPD rats exhibited damaged alveolar septa, alveolar enlargement and inflammatory cell infiltration (Fig. 1e and f). Collectively these data indicate that establishment of the COPD rat model was successful.

### Quadriceps muscle weight, cross-sectional area and fiber type

**Fig. 2:**
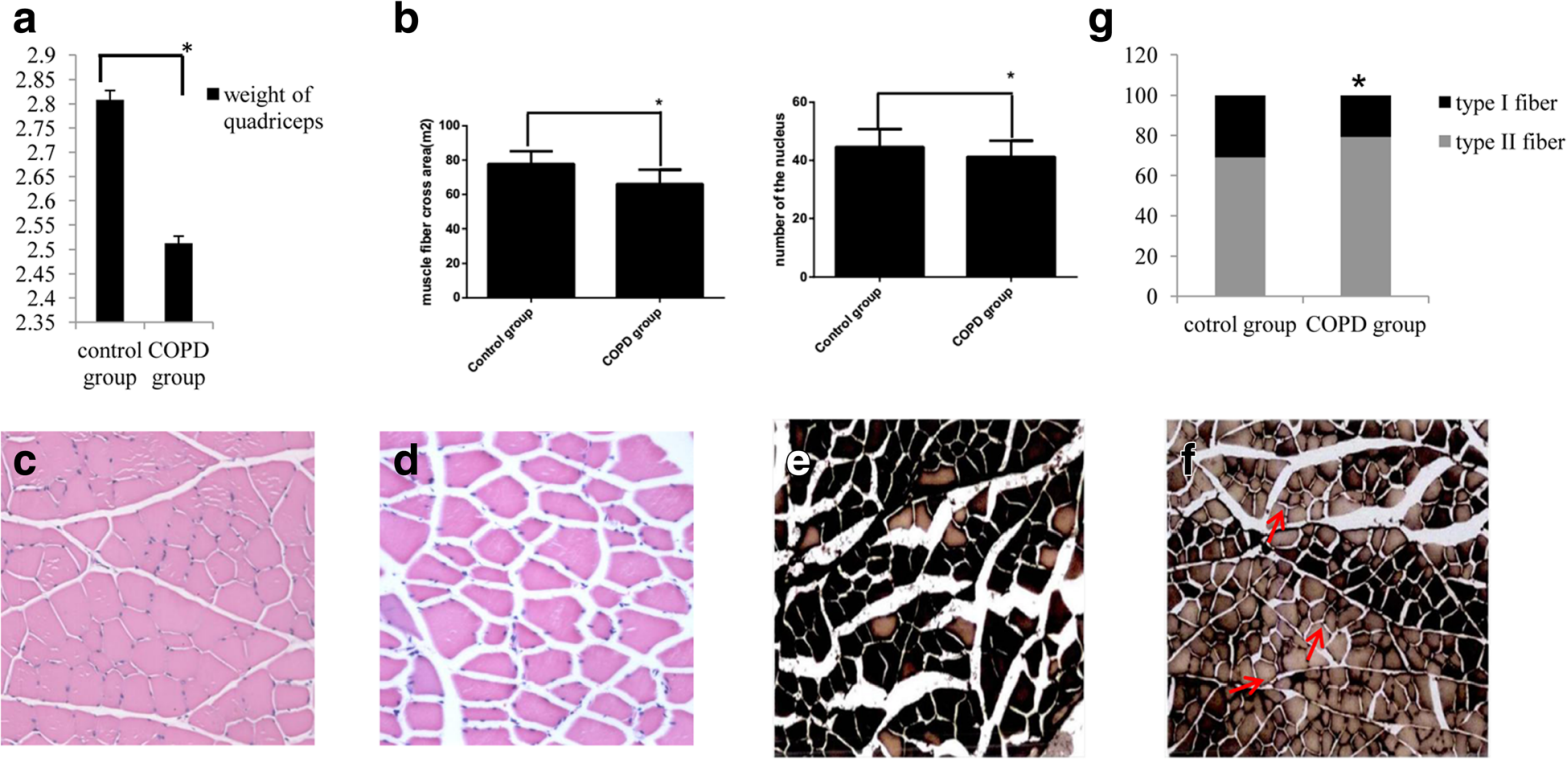
Alterations in the weight, cross-sectional area and fiber-type of the quadriceps in rats with COPD. Relative to controls, the **a** weight and **b** muscle fiber cross-sectional area and nuclear cell number of the quadriceps muscle in COPD rats were significantly reduced. n = 10, *P < 0.05,versus the control group. **c** Hematoxylin and eosin staining of quadriceps samples from the control group identified neat and tight muscle fibers, a narrow gap between adjacent muscle fibers, normal structures, a normal nucleus number and an even distribution (magnification, ×200). **d** Hematoxylin and eosin staining of quadriceps samples from the COPD group identified muscle fiber atrophy, disordered fiber arrangement, a wide gap between adjacent muscle fibers, a reduced number of nuclei and an uneven distribution of fiber sizes (indicated by black arrows; magnification, ×200) **e** ATPase histochemical staining (calcium-cobalt method) of quadriceps samples from the control group (magnification, ×100). Following staining, type I muscle fibers appear lighter in color than type II fibers. Control samples exhibited a larger proportion of type I muscle fibers. **f** ATPase histochemical staining of quadriceps samples from the COPD group (magnification, ×100). Type II fibers comprised a larger proportion of the total fibers in COPD samples (indicated by red arrows). **g** Quantification of muscle fiber proportions. Type I fibers were significantly decreased and type II fibers were significantly increased in the COPD group. n = 10, *P < 0.05,versus the control group. COPD, chronic obstructive pulmonary disease

Relative to control rats, COPD rats exhibited significant reductions in the weight (2.8028 ± 0.0195 vs. 2.5131 ± 0.0147 g, *P* < 0.05; Fig. 2a), muscle fiber cross-sectional area (77.7860 ± 7.4126 × 10^−4^ vs. 66.1556 ± 8.3279 × 10^−4^ m^2^, P < 0.05; Fig. 2b) and muscle fiber nuclear cell number (44.63 ± 6.16 vs. 41.23 ± 5.56; *P* < 0.05; Fig. 2b) of the quadriceps muscle. Histological analysis of quadriceps muscle samples obtained from control rats (Fig. 2c) identified neat and tight muscle fibers, narrow gaps between adjacent muscle fibers and an even distribution. By contrast, those obtained from COPD rats exhibited muscle fiber atrophy and a disordered fiber arrangement, a wide gap between adjacent muscle fibers, a reduction in nucleus number and an uneven size distribution (Fig. 2d). ATPase histochemistry (calcium-cobalt method) also indicated that the muscle fiber type of COPD model rats was altered (Fig. 2e–g). Relative to controls, the type I fiber content of COPD rats significantly decreased (30.9 ± 6.6 vs. 20.7 ± 7.6%, respectively, *P* < 0.05) and the type II fiber content significantly increased (79.3 ± 7.6% vs. 30.9 ± 6.6%, respectively, *P* < 0.05; Fig. 2g).

### Correlation of TWEAK expression with quadriceps muscle weight and fiber type

**Fig. 3:**
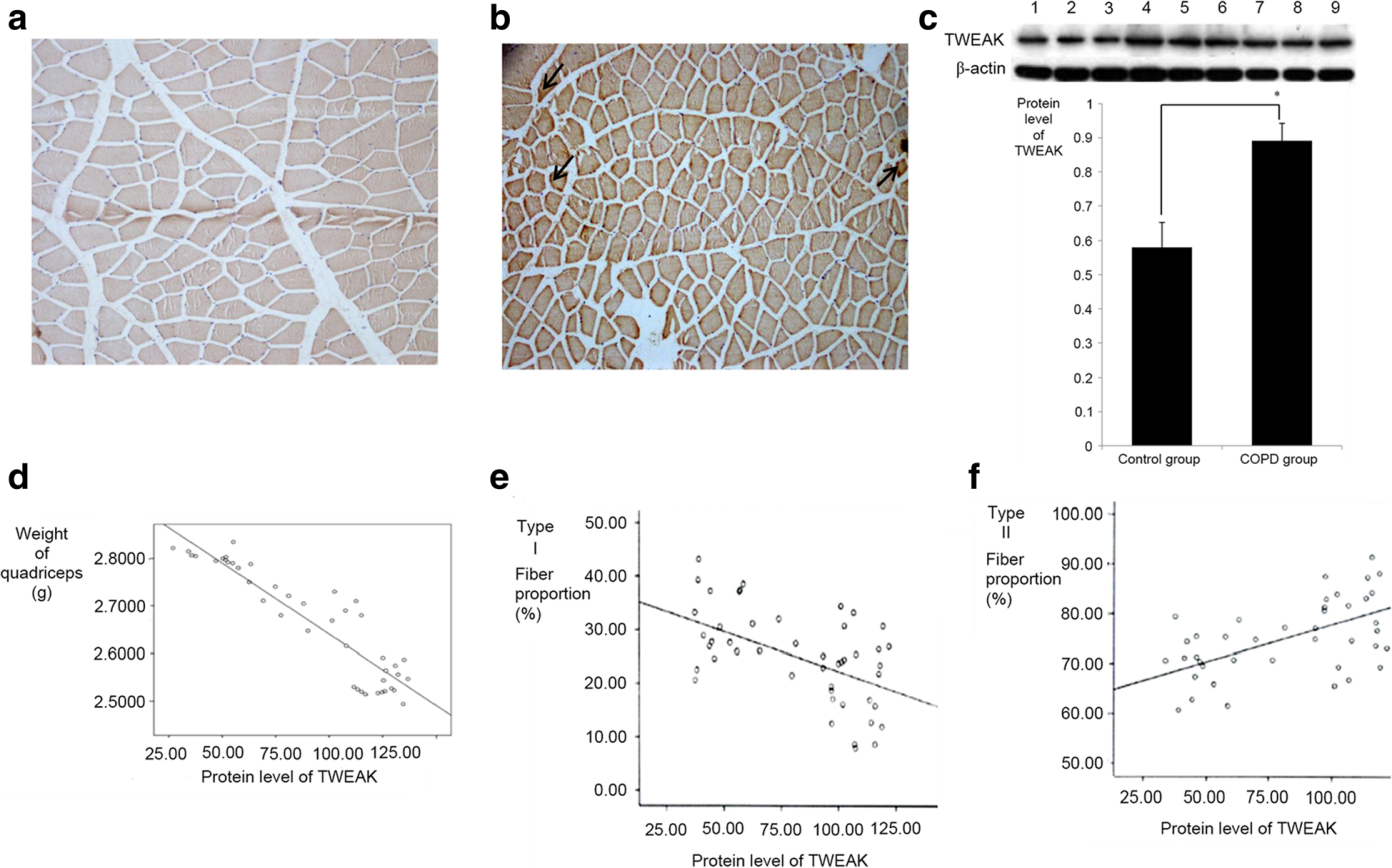
Correlation of TWEAK expression with quadriceps weight and fiber type. Immunohistochemical staining of TWEAK expression in the quadriceps muscle of **a** control and **b** COPD model rats. Markedly increased TWEAK expression was observed in the COPD group. TWEAK was concentrated to the membrane of the skeletal muscle cells (brown stain, black arrows; magnification, ×100). **c** Western blot analysis of TWEAK expression. Lanes 1–3 represent the control group and lanes 4–9 represent the COPD group. Levels of TWEAK expression were significant higher in COPD samples. *n* = 6, *P < 0.05,versus the control group. **d** Correlation between TWEAK expression and quadriceps muscle weight. **e** Correlation between TWEAK expression and type I fiber proportion. **f** Correlation between TWEAK and type II fiber proportion. TWEAK expression was significantly proportional (^*^ *P* < 0.05) to the type II fiber ratio while being significantly inversely proportional to the weight and type I fiber proportion of the quadriceps (^*^ *P* < 0.05). TWEAK, tumor necrosis factor-like weak inducer of apoptosis; COPD, chronic obstructive pulmonary disease

TWEAK drives different physiological effects, including cell proliferation, differentiation, angiogenesis, migration and apoptosis [28]. In the current study, it was observed that TWEAK localized to the membrane of quadriceps muscle cells (Fig. 3a and b). The presence of TWEAK was indicated by varying degrees of tan or brown granules or by the deposition of flakes, identified by analyzing pathological images of quadriceps muscle sections with Image-Pro Plus 6.0 software. Relative to control rats (Fig. 3a), it was observed that levels of TWEAK expression in the quadriceps muscle of COPD rats (Fig. 3b) were markedly increased. TWEAK expression was also measured by western blot analysis, whereby it was observed that levels of TWEAK in the COPD model group were significantly higher than that in the control group (0.8910 ± 0.0512 vs. 0.5803 ± 0.0733, respectively, *P* < 0.05; Fig. 3c). In addition, levels of TWEAK expression were inversely proportional to muscle weight and type I fiber proportion (*P* < 0.05; Fig. 3d and e), and were proportional to the ratio of type II fibers in quadriceps muscle (*P* < 0.05; Fig. 3f).

### Expression of NF-κB, MuRF1 and PGC-1α in the quadriceps muscle

**Fig. 4.**
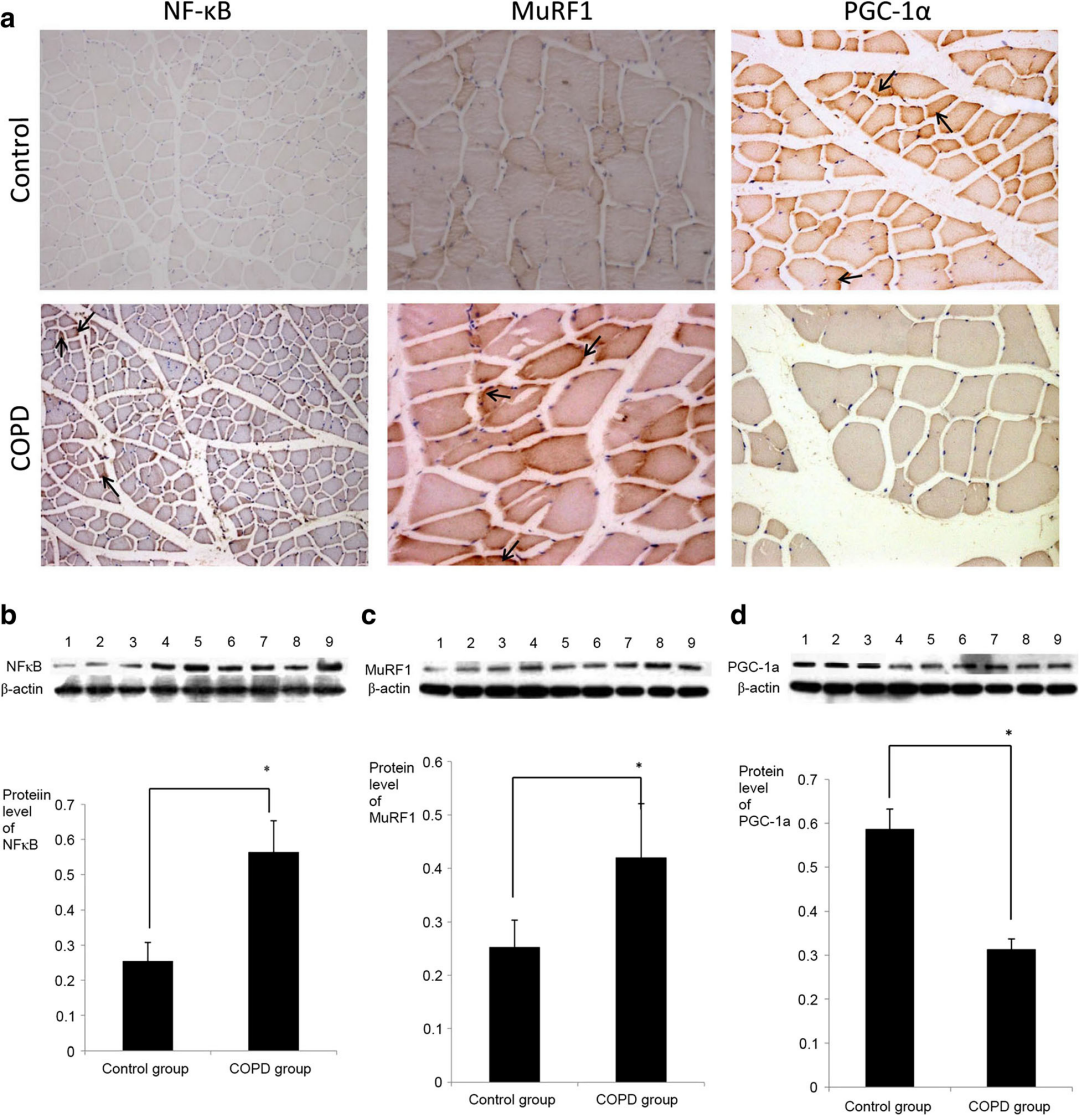
Expression of NF-κB, MuRF1 and PGC-1α in the quadriceps of COPD rats. **a** Immunohistochemical staining of NF-κB, MuRF1 and PGC-1α in quadriceps samples from control and COPD model rats (NF-κB magnification, ×100, MuRF1 and PGC-1α magnification, ×200). Samples from COPD rats exhibited higher levels of NF-κB and MurF1 and lower levels of PGC-1α, relative to controls. All three factors were localized to the membranes of skeletal muscle cells (black arrows). Subsequent western blot analysis indicated that the changes in **b** NF-κB, **c** MuRF1 and **d** PGC-1α protein expression were significant. Lanes 1–3 represent the control group and lanes 4–9 represent the COPD group. n = 6, *P < 0.05,versus the control group. COPD, chronic obstructive pulmonary disease; NF-κB, nuclear factor-κB; MuRF1, muscle ring finger-1; PGC-lα, peroxisome proliferator-activated receptor-γ coactivator-lα

A previous study by the present authors demonstrated that the levels of NF-κB were increased in the peripheral skeletal muscle of COPD rats, where it potentially serves a key role in the pathogenesis of COPD (1). In the current study, immunohistochemical staining and western blot analysis were used to measure the levels of NF-κB and its potential downstream effectors MuRF1 and PGC-1α. Relative to controls, immunohistochemical staining identified altered expression in all three proteins in COPD rats (Fig. 4a). This was confirmed by western blotting, whereby significant increases in NF-κB and MuRF1 (both *P* < 0.05) and significant reductions in PGC-1α (P < 0.05) were detected (Fig. 4b–d).

## Discussion

The current study established a COPD rat model based on three parameters: weight, lung function and lung histomorphology. In comparison with control rats, the body weights of rats with COPD increased at significantly slower rates, and the PEF, FEV0.3 and FEV0.3/FVC of COPD rats were significantly reduced. Furthermore, lung tissue samples from controls exhibited thin alveolar septa, normal alveoli and no inflammatory cell infiltration, whereas those obtained from COPD rats exhibited destruction of the alveolar septa, alveolar enlargement and inflammatory cell infiltration. These results indicated that a COPD rat model had been successfully established.

Muscle mass depletion is exhibited by 30–40% of individuals with COPD [38]. Patients with COPD may also experience malnutrition, typically due to skeletal muscle atrophy [39], which in turn may be a useful prognostic indicator of remission and mortality [40, 41]. In addition to a slower rate of body weight increase, the current study observed that the weight, muscle fiber cross-sectional area and muscle fiber nuclear cell number of the quadriceps muscle were significantly reduced and exhibited morphological differences. These changes were analogous to those that accompany muscle mass depletion in patients with COPD. The quadriceps muscle obtained from rats with COPD also exhibited muscle fiber atrophy and disordered fiber arrangement, a wide gap between adjacent muscle fibers and an uneven fiber-size distribution. In a previous large-sample study of patients with severe to moderate COPD [42], it was observed by computer tomography that the mid-thigh cross-sectional area was a stronger predictor of mortality than lung function (FEV1). Similarly, it has been demonstrated that fat-free mass, but not fat mass, may be an independent predictor of survival rate, whereas atrophy may be a predictor of physical function and mortality rate [43]. Structural changes associated with COPD include decreases in the proportion and size of type I fibers in the quadriceps muscle [39], which were confirmed in the present study. A previous meta-analysis of 84 patients with COPD from eight studies demonstrated that proportions of type I and type IIX fibers of <27% and >29%, respectively, in the vastus lateralis may be defined as pathological [44], suggesting that patients with COPD exhibit a reduction in type I fibers that is associated with disease severity. Therefore, alterations in fiber proportion may serve an important role in the pathogenesis of COPD.

TWEAK is a member of the TNF super family of cytokines and in a similar way to TNF-α, is initially synthesized as a type II transmembrane protein (249 amino acids) [45, 46]. However, membrane-bound TWEAK is cleaved into a soluble form (156 amino acids) by furin, which is a calcium-dependent serine endoprotease [45–47]. Specific atrophic conditions, including denervation, immobilization and starvation, promote TWEAK signaling and lead to skeletal muscle atrophy. TWEAK is also responsible for a slow-to-fast-type fiber transition within skeletal muscle [20, 47]. In the present study, TWEAK expression was significantly increased in the quadriceps muscle of COPD model rats and was associated with the weight and fiber type alternation of the muscle. These findings suggest that TWEAK may be a key regulator of quadriceps muscle atrophy in COPD, though its underlying mechanisms of action remain unknown.

Several factors and mechanisms have been implicated in the etiology of COPD muscle dysfunction, and oxidative stress, systemic inflammation, structural abnormalities, mitochondrial derangements, autophagy, muscle wasting, and deconditioning are considered to be the primary biological contributors [5–17]. In addition, type II muscle fibers are more susceptible to atrophy than type I fibers in many chronic diseases [1, 2]. The present study demonstrated that type I fibers were decreased while type II fibers were increased in the COPD group. Furthermore, fiber-type alterations were correlated with TWEAK expression. Future studies are required in TWEAK-Tg mice to determine whether slow-to-fast-type fiber transitions and quadriceps muscle atrophy are essential for TWEAK-induced atrophy in COPD. Previous results have suggested that overexpression of PGC-1α may inhibit TWEAK-induced atrophy, NF-κB activation and MuRF1 expression in cultured myotubes [28, 39]. Furthermore, progressive muscle atrophy observed in TWEAK-Tg mice is significantly attenuated in TWEAK-PGC-1α double Tg mice, suggesting that PGC-1α serves a key role in TWEAK-induced muscle atrophy [39]. In the present study, levels of NF-κB, MuRF1 and PGC-1α were abnormal in rats with COPD, thus suggesting that these factors serve regulatory roles in COPD-related atrophy. Therefore, in COPD rats, TWEAK may activate NF-κB and subsequently cause alterations in the atrophy and fiber transition of the quadriceps muscle through MuRF1 and PGC-1α. However, the underlying mechanisms to confirm this hypothesis remain unknown. Future cell and molecular biology studies are required to determine the underlying mechanisms of TWEAK regarding its effects on quadriceps muscle atrophy, fiber-type alteration and the expression of MuRF1, NF-κB and PGC-1α, with the latter also requiring confirmation in patients with COPD. In particular, studies into the effects of cigarette smoke exposure in TWEAK knockout mice may aid to determine the roles of TWEAK in COPD-induced muscle wasting.

The current study used cigarette smoke to establish a COPD model in rats. Smoking is also a primary cause of COPD in human patients, possibly due to its stimulatory effects on oxidative stress and systemic inflammation. However, it is unclear whether smoking has an influence on the expression of TWEAK, as a prerequisite for the downstream effects of TWEAK on quadriceps muscle atrophy and fiber-type alteration. Thus, further studies are required to assess the impact of smoking on TWEAK expression.

## Conclusion

In conclusion, the present study in COPD model rats identified secondary dysfunction of the peripheral muscles, characterized by muscular atrophy and alterations in fiber type and composition. In addition, TWEAK expression was significantly increased in the quadriceps of COPD rats, and may play important role in the atrophy and fiber-type alteration of the quadriceps muscle. Clinical consequences of these muscular alterations include impaired exercise tolerance, low physical activity and a decreased quality of life for patients with COPD. It was also indicated that the effects of TWEAK are possibly due to its regulation of PGC-1α and MuRF1, and thus these factors may be potential targets in the treatment of muscle atrophy in COPD. The inhibition of TWEAK may also be a potential therapy for the maintenance of skeletal muscle mass and metabolic function during COPD. Therefore, the TWEAK system may be a primary drug target for the treatment of muscle atrophy in COPD.

## Abbreviations

COPD: Chronic obstructive pulmonary disease
Fnl4: Fibroblast growth factor-inducible 14
MAFbX: Muscle atrophy F-Box
MuRF1: Muscle ring finger-1
NF-κB: Nuclear factor-κ B
PGC-lα: Peroxisome proliferator-activated receptor-γ coactivator-lα
TWEAK: Tumor necrosis factor-like weak inducer of apoptosis
